# Thermal Robustness of Entanglement in a Dissipative Two-Dimensional Spin System in an Inhomogeneous Magnetic Field

**DOI:** 10.3390/e23081066

**Published:** 2021-08-17

**Authors:** Gehad Sadiek, Samaher Almalki

**Affiliations:** 1 Department of Applied Physics and Astronomy, University of Sharjah, Sharjah 27272, United Arab Emirates; 2 Department of Physics, Ain Shams University, Cairo 11566, Egypt; 3 Department of Physics and Astronomy, King Saud University, Riyadh 11451, Saudi Arabia; sam24241@hotmail.com

**Keywords:** quantum entanglement, quantum information, dissipative environment, quantum spin systems

## Abstract

Recently new novel magnetic phases were shown to exist in the asymptotic steady states of spin systems coupled to dissipative environments at zero temperature. Tuning the different system parameters led to quantum phase transitions among those states. We study, here, a finite two-dimensional Heisenberg triangular spin lattice coupled to a dissipative Markovian Lindblad environment at finite temperature. We show how applying an inhomogeneous magnetic field to the system at different degrees of anisotropy may significantly affect the spin states, and the entanglement properties and distribution among the spins in the asymptotic steady state of the system. In particular, applying an inhomogeneous field with an inward (growing) gradient toward the central spin is found to considerably enhance the nearest neighbor entanglement and its robustness against the thermal dissipative decay effect in the completely anisotropic (Ising) system, whereas the beyond nearest neighbor ones vanish entirely. The spins of the system in this case reach different steady states depending on their positions in the lattice. However, the inhomogeneity of the field shows no effect on the entanglement in the completely isotropic (XXX) system, which vanishes asymptotically under any system configuration and the spins relax to a separable (disentangled) steady state with all the spins reaching a common spin state. Interestingly, applying the same field to a partially anisotropic (XYZ) system does not just enhance the nearest neighbor entanglements and their thermal robustness but all the long-range ones as well, while the spins relax asymptotically to very distinguished spin states, which is a sign of a critical behavior taking place at this combination of system anisotropy and field inhomogeneity.

## 1. Introduction

Quantum entanglement is considered to be the physical resource responsible for manipulating the linear superposition of the quantum states in many body quantum systems [1]. Entanglement, and its derivatives, show scaling behavior as the quantum system crosses a quantum phase transition critical point [2]. In particular, it is crucial in quantum information processing fields such as quantum teleportation, cryptography, and quantum computation [3]. However, quantum entanglement is very fragile due to the induced decoherence caused by the inevitable coupling of the quantum system to its surrounding environment [4,5]. The main effect of decoherence is to randomize the relative coherent phases of the possible states of the quantum system diminishing its quantum aspects. As a result, it is considered to be one of the main obstacles toward realizing an effective quantum computing system. The decoherence in the system sweeps out entanglement between the different parties of the system. Therefore, creating, quantifying, transferring and protecting entanglement in quantum states of many body systems are in the focus of interest of both theoretical and experimental research. Many of the newly engineered quantum systems that are considered promising candidates for the underlying technology of quantum information processing, such as cold atoms in optical lattices, ultracold atoms, optical microcavities, trapped ions and superconducting circuits [6,7,8,9,10,11,12,13], represent great experimental framework for studying dissipative effects in driven many-body quantum systems. On the other hand, the Heisenberg interacting spin systems have been in focus of interest for their own sake as they describe the novel physics of localized spins in magnetic systems as well as for their successful role in modeling many of these new customized physical systems. Moreover, many of these new systems can be used to simulate Heisenberg spin systems in a highly controllable manner.

Entanglement properties and dynamics in Heisenberg spin chains in absence of dissipative environments have been studied intensively [14,15,16,17,18,19,20,21,22,23,24]. The dynamics of a system of interacting qubits, represented by Heisenberg spin model, coupled to a dissipative environment has been studied in many works as well. The problem of two interacting qubits coupled to a dissipative environment has been investigated both analytically and numerically [25,26,27]. The one-dimensional interacting spin chains, N>2, coupled to dissipative environments were investigated as well at different degrees of anisotropy, magnetic field strength and temperatures [28,29,30,31,32,33,34,35]. The dynamics of entanglement in the Ising and isotropic (XXX) one-dimensional spin chains has been studied [30,33] through a numerical stochastic approach using the quantum state diffusion theory [36], to overcome the problem of the huge needed storage space. In an interesting relevant work, a one-dimensional chain of superconducting Josephson qubits with realistic values was studied under the effect of a noisy and disorder environment [29]. The noise effect was introduced as a set of bosonic baths, where each one of them was coupled to a separate qubit. the system was described as an Ising spin chain coupled to a Markovian environment after tracing out the bath degrees of freedom, which evolves asymptotically to steady state. In a previous work we have studied the entanglement dynamics in a one-dimensional spin chain in an external homogeneous magnetic field coupled to a Markovian dissipative environment. We showed how the interplay among the different system parameters can control the time evolution and asymptotic steady state of the system [37].

Recently there has been great interest in studying unconventional magnetism in spin systems in the absence and presence of dissipative effects, where new non-traditional magnetic phases emerged as a result of varying the system key parameters such as the system anisotropy and the inhomogeneity of the external magnetic field [38,39,40,41,42,43,44,45,46,47,48,49]. While the ground state properties of the system were found to dictate its behavior in the equilibrium critical phenomena at zero temperature, the asymptotic steady state density matrix was the major player in the presence of dissipative effects. In a pioneering work, Lee et al. studied an anisotropic XYZ Heisenberg system of localized spins on a d-dimensional lattice at zero temperature under dissipative spin-flip process, associated with optical pumping. They showed how the asymptotic behavior of the system can exhibit new novel magnetic phases, as the degree of anisotropy of the spin-spin interaction is varied in the absence of external magnetic fields [38]. The impact of an inhomogeneous magnetic field on entanglement and coherence in a closed system of a pair of XXZ interacting *s* spins was investigated at zero and finite temperature [39]. The critical behavior of the system and its different phases at different values of the spin (s≥1/2), field gradient, spin interaction anisotropy and temperature were investigated. It was demonstrated how the inhomogeneity of the magnetic field can be used to control the system energy eigenlevels and enhance its entanglement content. Additionally, it was shown that the limiting temperature of entanglement in this system is mainly decided by the magnetic field gradient, where at a certain temperature, the system becomes entangled above a threshold value of that gradient. A finite XXZ systems of arbitrary spin under inhomogeneous fields were studied too [50], where it was shown that highly degenerate exactly separable symmetry breaking ground states can be obtained for a wide range of inhomogeneous field configurations of zero sum in arrays of any dimension. Recently, the dissipative phase transition of an anisotropic XYZ Heisenberg spin-1/2 system in a finite two-dimensional lattice, at zero temperature, was investigated [51], by applying the corner-space renormalization method [52]. The linear response of the system was studied under the effect of an applied polarizing magnetic field in the *xy*-plane and subject to a dissipative incoherent spin relaxation process. The finite size scaling of the susceptibility of the system was carried out, which exhibited a critical behavior, where its peak value increased as a power law of the system size. The finite size scaling of the quantum fisher information indicated a critical behavior of the entanglement at the transition point. Very recently, spin-*s* chains with ferromagnetic XXZ coupling in the presence of sparse alternating magnetic fields have been studied. The exact ground state of the system was investigated and was found to exhibit a non-trivial magnetic behavior, where it shows significant magnetization plateaus sustainable at large system size [53].

Frustrated many-body quantum systems presents a very rich framework for studying exotic phenomena, which significantly rely on the entanglement properties, dynamics and sharing in the system [54]. A well-known system that presents such frustration is the Heisenberg spin model on a triangular lattice. The geometry of the triangular lattice is the mean reason behind this frustration, which leads to a highly degenerate ground state of the system. Such a geometric frustration has been experimentally realized in optical lattices [55], trapped ions [56] and magnetic nano-structures [57], which all have a great potential for applications in quantum simulation, computations and logic gates. In an interesting pioneering work, Cai et al. [58] proposed a scalable architecture for a practically realizable large-scale quantum simulator at room temperature. This architecture is based on a triangular (rectangular) lattice of interacting spins that can be fabricated chemically on a fluorine-terminated diamond surface. They demonstrated that this system can be used efficiently to simulate quantum spin models with tunable spin-spin interaction. They used an external magnetic field to tune the effective spin-spin interaction and also to force a magnetic phase transition. As can be concluded form the literature review, the dynamics and asymptotic behavior of a two-dimensional spin lattice has not been studied under the combined effect of a thermal dissipative environment and an inhomogeneous magnetic field yet.

In this paper, we study the time evolution and the asymptotic steady state of the bipartite quantum entanglement and spin relaxation in a finite two-dimensional Heisenberg spin-1/2 triangular lattice, where a single central spin is surrounded by equally distant spins, with nearest-neighbor spin interaction under the influence of a dissipative Lindblad environment at zero and finite temperatures. We investigate the impact of an external inhomogeneous magnetic field on the entanglement sharing, dynamics, asymptotic behavior and robustness against the thermal dissipative effect of the environment. We show how a particular inhomogeneous magnetic field setup, where the gradient is directed toward the central spin, can significantly enhance the bipartite entanglement among the nearest neighbor spins and boost their thermal robustness in the completely anisotropic (Ising) system and even the beyond nearest neighbors in the partially anisotropic system, which indicates that a long range quantum correlation is taking place across the lattice at this combination of inhomogeneity of the magnetic field and anisotropy of spin-spin coupling. Additionally, we explore the associated spin dynamics and relaxation process as we vary the inhomogeneity of the field, the anisotropy of the spin interaction and the environment temperature. We demonstrate how the same particular inhomogeneous magnetic field setup has the strongest influence, compared with all other setups, on the steady state of the spins in the system except at zero anisotropy. We show how in the steady state of the system, the spins reach different states that are most distinguished from each other in the partially anisotropic system accompanying the long rang quantum correlation indicating a tendency of this finite system to exhibit a critical behavior.

This paper is organized as follows. In the next section, we present our model and density matrix calculations. In Section 3 and Section 4, we study the time evolution of entanglement and the spin relaxation respectively, in the spin system at different degrees of anisotropy. We conclude in Section 5.

## 2. The Model

**Figure 1:**
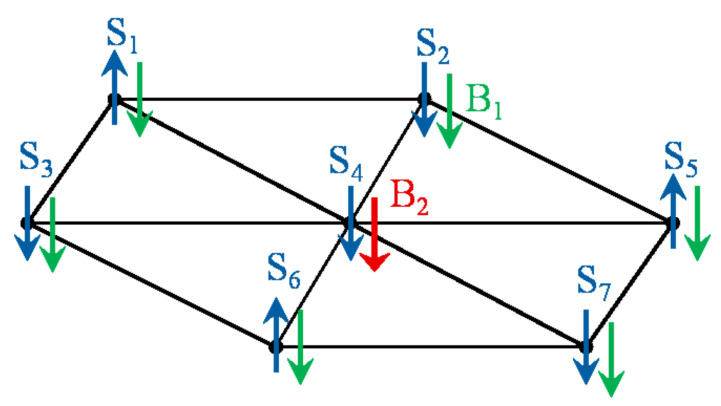
A two-dimensional triangular spin lattice in the presence of an external inhomogeneous magnetic field with strengths $B_{1}$ at the border sites and $B_{2}$ at the central one.

We consider a set of 7 localized spin-$\frac{1}{2}$ particles in a two-dimensional triangular lattice coupled through nearest neighbor exchange interaction *J* and subject to an external inhomogeneous magnetic field, as shown in Figure 1. The Hamiltonian of the system is given by

(1) $$H=\frac{\left(1+\gamma \right)}{2}J\sum_{\langle i,j\rangle }^{N}S_{i}^{x}S_{j}^{x}+\frac{\left(1-\gamma \right)}{2}J\sum_{\langle i,j\rangle }^{N}S_{i}^{y}S_{j}^{y}+\delta J\sum_{\langle i,j\rangle }^{N}S_{i}^{z}S_{j}^{z}+\sum_{i=1}^{N}h_{i}^{z}S_{i}^{z}\;,$$

where: $S_{i}^{\alpha }$ = $\frac{1}{2}$$\sigma_{i}^{\alpha }$ (α = x, y or z) and $\sigma_{i}^{\alpha }$ are the local spin-$\frac{1}{2}$ operators and Pauli operators, respectively, for convenience, we set ℏ=k=1. γ and δ are the anisotropy parameters, which determines the relative strength of the spin-spin coupling in the *x*, *y* and *z*-directions. We study different classes of the Heisenberg spin system, by changing the values of γ and δ, such as the Ising (γ=1 and δ=0), XXX (γ=0 and δ=0.5), XYZ (γ=0.5 and δ=1), etc. The system is subject to an external inhomogeneous static magnetic field applied in the z-direction such that the magnetic field strength at the border sites is $h_{i}=B_{1}\;\left(i=1,2,3,5,6,7\right)$, whereas the strength at the central site is $h_{4}=B_{2}$. We assume that the maximum external magnetic field strength is ω.

The dynamics of an isolated quantum system is described by the time evolution of its density matrix ρ(t) according to the quantum Liouville equation $\dot{\rho }\left(t\right)=-i\left[H,\rho \right]$. For an open quantum system interacting with its environment, where the system and the environment satisfy the Born-Markovian approximation, the time evolution of the system is best described by the Lindblad Master equation [59,60], which is defined as

(2) $$\dot{\rho }\left(t\right)=-i\left[H,\rho \right]+D_{\rho }\;,$$

where $D_{\rho }$ is the extra term that describes the dissipative dynamics and is represented in the Lindblad form as

(3) $$D_{\rho }=-\frac{1}{2}\sum_{j=1}^{M}\sum_{k=1}^{N}\left\{\left[L_{k}^{\left(j\right)}\rho ,L_{k}^{(j)†}\right]+\left[L_{k}^{\left(j\right)},\rho L_{k}^{(j)†}\right]\right\}\;,$$

where the Lindblad operator $L_{k}^{\left(j\right)}$ represents the effect of the considered environment on the system site *k*, and the environment is assumed to couple to each site independently of the other sites, *M* is the total number of Lindblad operators and *N* is the total number of sites. It is more convenient to work in the Liouville space, where the density operator is represented as a vector. As a result, Equation (2) can be recasted into the matrix equation form

(4) $$\vec{\dot{\rho }}\left(t\right)=\left({\hat{L}}^{H}+{\hat{L}}^{D}\right)\vec{\rho }=\hat{L}\vec{\rho }\;,$$

where ${\hat{L}}^{H}$ and ${\hat{L}}^{D}$ are superoperators acting on the vector ρ in the Liouville space, where the first one represents the unitary evolution due to the free Hamiltonian while the second represents the dissipation process.

The matrix elements of $\dot{\rho }$ are defined as

(5) $${\dot{\rho }}_{jl}\left(t\right)=-i\sum_{m,n}\;\;\left(L_{jl,mn}^{H}+L_{jl,mn}^{D}\right)\;\;\rho_{mn}\;,$$

where the tetrahedral matrices $L^{H}$ and $L^{D}$ are given by

(6) $$L_{jl,mn}^{H}=H_{jm}\delta_{ln}-\delta_{jm}H_{nl}\;,$$

and

(7) $$L_{jl,mn}^{D}=\frac{i}{2}\sum_{k}\left[2{\left(L_{k}^{†}\right)}_{nl}{\left(L_{k}\right)}_{jm}-{\left(L_{k}^{†}L_{k}\right)}_{jm}\delta_{ln}-\delta_{jm}{\left(L_{k}^{†}L_{k}\right)}_{nl}\right]\;.$$

A detailed discussion of these theoretical arguments was provided in our previous work, Ref. [37]. The solution of Equation (4) yields the density vector as

(8) $$\vec{\rho }\left(t\right)=\sum_{i}A_{i}\;{\vec{\eta }}_{i}\;e^{\lambda_{i}\;t}\;,$$

where the coefficients $A_{i}$ are determined from the initial conditions of the evolution process, $\left\{\lambda_{i}\right\}$ and $\left\{{\vec{\eta }}_{i}\right\}$ are the sets of all eigenvalues and eigenvectors of the tetrahedral matrix L. As can be noticed, we have converted the Lindblad master equation, Equation (2), of the system into a matrix equation, Equation (4), by transforming the problem to the Liouville space. Then by exact numerical diagonalization of the matrix $\hat{L}$, which is a very hard task, one obtains the set of eigenvalues and eigenvectors needed to find the evolved density matrix ρ(t). For a two-dimensional system with *N* spin-1/2 particles, the dimension of the Hilbert space is $2^{N}$ and the dimension of the tetrahedral matrices is $2^{2N}$ which, even for a small number of spins, is extremely large. For an exact numerical treatment of the problem, one needs to store $2^{4N}$ matrix elements, of the matrix L, before being able to diagonalize the matrix, which is an enormous number, even for a few spins. For our considered system, N=7, we needed to store $2^{28}$ matrix elements, i.e., more than a quarter of a billion. As a result of these huge dimensions, it is practically impossible to provide an analytical treatment of the problem and is even cumbersome to do it numerically. Adding only one extra spin will make the matrix elements exceed 4 billion, which is more than the available memory in most of the current computing systems. As a result, many different approaches rely on approximate treatment of the system dynamics such as the numerical stochastic technique, which applies the quantum state diffusion theory [36] or the corner space renormalization method [52]. To study the next symmetric triangular lattice, we need to add 12 spins, which is beyond the ability of any existing supercomputing system to handle. Therefore, testing the effect of the system size, using the exact numerical technique, is a quite hard task, particularly while preserving the symmetry of the two-dimensional lattice.

For the considered spin system, the effects of thermal relaxing and exciting environment, respectively, are represented by the two operators

(9) $$L_{k}^{\left(1\right)}=\Gamma \;\left(\bar{n}+1\right)\;S_{k}^{-},\;\;\;L_{k}^{\left(2\right)}=\Gamma \;\left(\bar{n}\right)\;S_{k}^{+}\;\;\;\;\;\;\;\left(k=1,2,\cdots ,N\right),\;$$

where $S_{k}^{+}$ and $S_{k}^{-}$ are the spin raising and lowering operators, $S_{k}^{\pm }$ = $S_{k}^{x}$ ± $iS_{k}^{y}$. The quantity $\bar{n}$, which reflects the mean number of excitations in the environment, accounts for the thermal influence of the environment and is proportional to its temperature, whereas Γ is a phenomenological parameter that represents the interaction strength between the quantum system and the environment, which determines the decay rate [29,60,61]. Accordingly, the dissipation part of the master equation, Equation (2), represents spontaneous emission as well as thermally induced absorption and emission processes [60].

We adopt the concurrence as a measure of the bipartite entanglement in the system, where Wootters [62] has shown that for a pair of two-state systems *i* and *j*, the concurrence $C_{i,j}$, which varies between 0 to 1, can be used to quantify the entanglement between them and is defined by $C_{i,j}\left(\rho_{i,j}\right)=max\left\{0,\epsilon_{1}-\epsilon_{2}-\epsilon_{3}-\epsilon_{4}\right\}$, where $\rho_{i,j}$ is the reduced density matrix of the two spins under consideration, $\epsilon_{i}$’s are the eigenvalues of the Hermitian matrix $R\equiv \sqrt{\sqrt{\rho_{i,j}}\tilde{\rho_{i,j}}\sqrt{\rho_{i,j}}}$ with $\tilde{\rho_{i,j}}=\left(\sigma^{y}⊗\sigma^{y}\right)\rho_{i,j}^{*}\left(\sigma^{y}⊗\sigma^{y}\right)$ and $\sigma^{y}$ is the Pauli matrix of the spin in the *y*-direction. We study the time evolution of the system using the standard basis $\left\{|↑↑\cdots ↑\rangle ,|↑↑\cdots ↓\rangle ,\cdots ,|↑↓\cdots ↓\rangle ,\cdots ,|↓↓\cdots ↓\rangle \right\}$ and starting from different initial typical states: a separable (disentangled) state, $|\psi_{s}\rangle =|↑↑\cdots ↑\rangle$; a partially entangled (*W*-state), $|\psi_{w}\rangle$=$\frac{1}{\sqrt{N}}\left(|↑↓\cdots ↓\rangle +|↓↑\cdots ↓\rangle +\cdots +|↓↓\cdots ↑\rangle \right)$ and a maximally entangled state, $|\psi_{m}\rangle =\frac{1}{\sqrt{2}}\left(|↑↓\rangle +|↓↑\rangle \right)|↓↓\cdots ↓\rangle$.

## 3. Dynamics of Entanglement

When a single spin-1/2 particle is inserted, at rest, into a homogeneous magnetic field, it precesses around the magnetic field direction with a constant angle that depends on the initial state of the spin, and with a (Larmor) frequency that is determined by the strength of the applied field. For a spin system with an XYZ nearest neighbor interaction, as described by the Hamiltonian (1), in absence of an external magnetic field $\left(h_{i}^{z}=0\right)$, every spin in the system experiences an effective net magnetic field due to the interaction with all its neighboring spins. This magnetic field forces the spin to precess about the field direction, where the precession strength of every spin depends on its location in the system and the degree of anisotropy, the higher the anisotropy in the system is, the stronger is the precession. When this system couples to the Lindblad environment at zero temperature, only the first term, $L_{k}^{\left(1\right)}$, in Equation (9) is active with a decay effect on the precessing spin that acts to align it into the negative *z*-direction, |↓〉. Turning on the temperature activates the second term, $L_{k}^{\left(2\right)}$, which acts to align the spin into the positive *z*-direction, |↑〉; however, its effect is much smaller than the first term, as can be noticed from Equation (9), especially at very low temperatures where the quantum character of the system is preserved. The asymptotic steady state of every spin and its entanglement to the other spins is determined by the interplay between the spin-spin interaction effective field, responsible for precession, and the dissipative environment decay effect. While the initial state of the system may affect the initial and intermediate dynamics of the system, the asymptotic behavior is independent of it, as we will show in our results. In the extreme case of a completely isotropic system, the spins don not precess at all around the effective field and the dissipative decay effect dominates, at zero temperature, forcing all the spins to point downwards, parallel to each other, leading to an asymptotic separable steady state with zero entanglement. At finite temperature, the spins in the steady state stay parallel but slightly deviates from the downward direction. Introducing anisotropy to the system enhances the precession process, which competes with the dissipative decay effect and makes the system evolve to a steady state with a finite entanglement, where each spin may end up in a different state from the others depending on its location in the lattice and the existing symmetry.

Applying an external homogeneous magnetic field to the spin system adds up to the effective magnetic field and impacts the precession process in a way that depends on its magnitude and direction. Applying an inhomogeneous magnetic field causes a big variance in the asymptotic behavior and entanglement of each spin compared with the others depending on the field gradient magnitude and direction, which changes the entanglement distribution and sharing among the spins depending on their locations. As mentioned before, for Equation (2) to represent a good approximation for the time evolution of the system, certain restrictions have to apply to the system parameters, the coupling parameter between the system and the environment Γ as well as the relaxation time scale of the environment dynamics should be small compared to that of the system dynamics manifested by the parameter ω representing the spin precession frequency around the *z*-axis. As a result, we consider values of Γ and *J* such that Γ and J<<ω, where we set Γ=J=0.05ω, ω=1, and the temperature parameter $0\leq \bar{n}\leq 0.1\;(∼$41mK).

### 3.1. Anisotropic Spin System (Ising Model)

We start by studying the two-dimensional completely anisotropic (Ising) spin system coupled to the dissipative environment. For convenience, we consider the time evolution of the system in terms of the dimensionless time T=ωt. We work in a system of units where ω=ℏ=1, therefore, the time *t* is in units of $\omega^{-1}$, $B_{1}$ and $B_{2}$ and Γ are in units of ω, whereas $\bar{n}$ is a dimensionless parameter.

For the rest of the paper we focus on three different combinations of the magnetic fields $B_{1}$ and $B_{2}$, namely (1,1), (1,0.1), and (0.1,1),which are represented in panels (a), (b) and (c) respectively in every figure in the paper, unless otherwise is stated explicitly. Additionally, we adopt a color code for the temperature parameter in all figures in this paper, where we use a blue (solid) line for $\bar{n}=0$, a green (dashed) line for $\bar{n}=0.001$, a red (dash-dotted) line for $\bar{n}=0.005$, a violet (dotted) line for $\bar{n}=0.01$, a black (solid with x marks) line for $\bar{n}=0.05$ and brown (dashed with x marks) line for $\bar{n}=0.1$, unless otherwise is stated explicitly.

**Figure 2:**
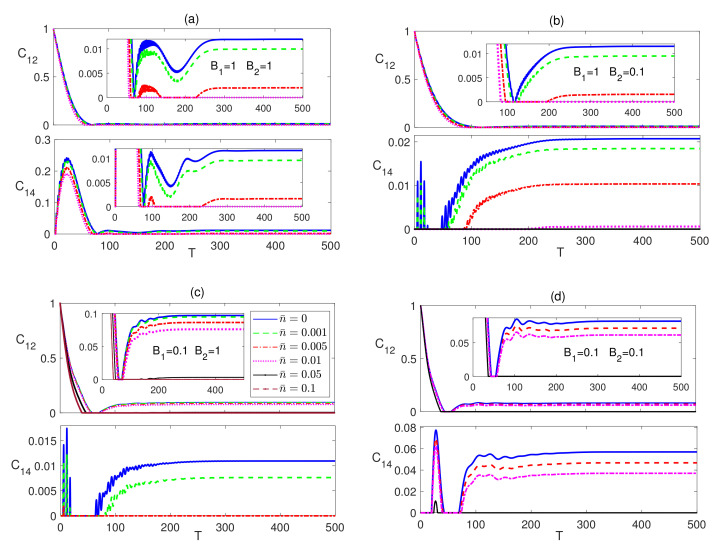
Time evolution of $C_{12}$ and $C_{14}$ in the Ising system in the presence of the environment (Γ=0.05) starting from an initial maximally entangled state at different temperatures ($0\leq \bar{n}\leq 0.1$) and different magnetic field strengths (**a**) $B_{1}=1$ and $B_{2}=1$, (**b**) $B_{1}=1$ and $B_{2}=0.1$, (**c**) $B_{1}=0.1$ and $B_{2}=1$ and (**d**) $B_{1}=0.1$ and $B_{2}=0.1$. The legend for all panels is as shown in panel (**c**). The time T=ωt is dimensionless, and for convenience we set ω=ℏ=1. $B_{1}$, $B_{2}$ and Γ are in units of ω, whereas $\bar{n}$ is a dimensionless parameter. The inner inset plots in the panels in this figure (and all other figures in this section) provide a magnifying look at the asymptotic behavior of the entanglement presented in the corresponding panels.

In Figure 2, in the upper panels, we depict the dynamical behavior of the nearest neighbor (nn) bipartite entanglement between the two border spins 1 and 2, $C_{12}$, starting from an initial maximally entangled state at different temperatures, where spins 1 and 2 are in a Bell state while all the other spins are in a separable state as described by $|\psi_{m}\rangle$. The inner inset plots in all panels in this paper provide a magnifying look at the asymptotic behavior of the entanglement. As can be noticed, in all the three magnetic field cases, $C_{12}$ starts at the maximum value and decays to zero before reviving again and asymptotically reaching a final steady state. The steady state entanglement value depends on both of inhomogeneity of the magnetic field and the environment temperature. Applying a non-homogeneous magnetic field where the border magnetic field is higher than the central one, $B_{1}>B_{2}$, slightly reduces the entanglement asymptotic value as shown in Figure 2b compared with that of the homogeneous case presented in Figure 2a at all temperatures. Clearly, raising the temperature is devastating to entanglement, where the entanglement value decreases significantly as the temperature increases until completely vanishing at around $\bar{n}=0.005$, as shown in Figure 2a. Figure 2b illustrates that in this inhomogeneous magnetic field case, entanglement is more fragile under the thermal effect. Interestingly, applying an inhomogeneous field with higher value at the center, $B_{1}<B_{2}$, as illustrated in Figure 2c, enhances the steady state entanglement significantly and makes it much more robust to the thermal excitation, where it persists up to $\bar{n}=0.1$, which is about 20 times higher than that of the other two cases, particularly at the border sites. The time evolution of the bipartite entanglement between the border spin 1 and the central spin 4, $C_{14}$, is explored in Figure 2, in the lower panels. The nn entanglement $C_{14}$ starts at zero before rising up to a maximum value then decaying again, vanishing for a period of time that increases with increasing temperature before reviving again to maintain an asymptotic steady state value. While the asymptotic value of $C_{14}$ is slightly lower than that of $C_{12}$ at all temperatures in the homogeneous case, as shown in Figure 2a, which is expected as a result of the entanglement sharing of the central spin with more nearest neighbor spins compared with spin 1. However, $C_{14}$ almost doubles when a weaker magnetic field is applied at the central spin compared with the border one with a higher robustness to temperature as illustrated in Figure 2b. Figure 2c shows that the asymptotic values of $C_{14}$ are lower than that of the homogeneous case when the stronger magnetic field is applied at the central spin, $B_{2}>B_{1}$. In Figure 2d, we test the effect of a weaker homogeneous magnetic field acting on the dissipative Ising system starting from an initial maximally entangled state. While this weaker magnetic field enhances the steady state values of both $C_{12}$ and $C_{14}$ compared with the observed values in Figure 2a, where a stronger homogeneous magnetic field was applied, still the inhomogeneous magnetic field, presented in Figure 2c, has a higher enhancement effect on the entanglement and thermal robustness, particularly, in the boarder sites.

**Figure 3:**
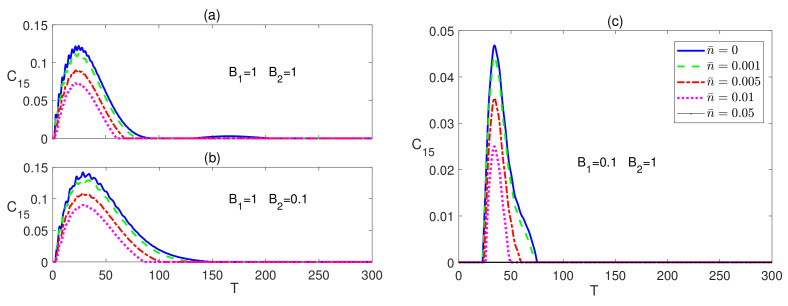
Time evolution of $C_{15}$ in the Ising system in the presence the of the environment (Γ=0.05) starting from an initial maximally entangled state at different temperatures ($0\leq \bar{n}\leq 0.05$) and different magnetic field strengths (**a**) $B_{1}=1$ and $B_{2}=1$, (**b**) $B_{1}=1$ and $B_{2}=0.1$, and (**c**) $B_{1}=0.1$ and $B_{2}=1$. The legend for all panels is as shown in panel (**c**).

**Figure 4:**
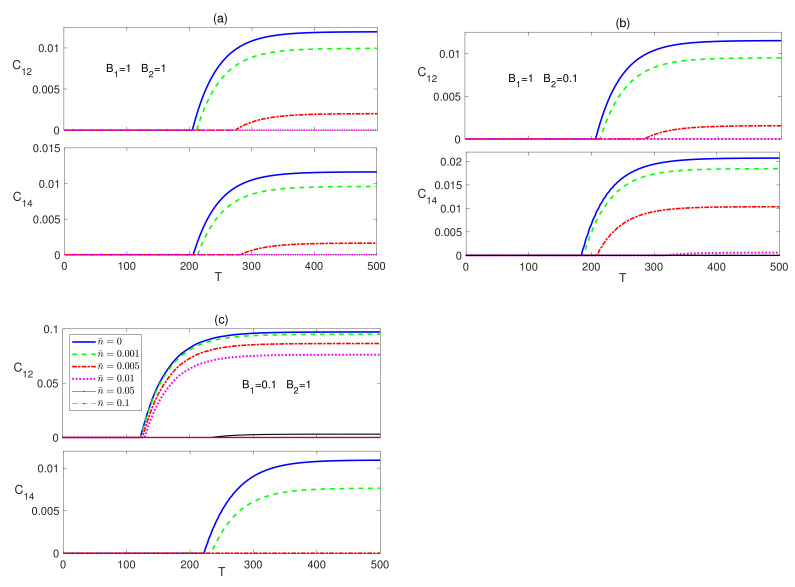
Time evolution of $C_{12}$ and $C_{14}$ in the Ising system in the presence of the environment (Γ=0.05) starting from an initial disentangled state at different temperatures ($0\leq \bar{n}\leq 0.1$) and different magnetic field strengths (**a**) $B_{1}=1$ and $B_{2}=1$, (**b**) $B_{1}=1$ and $B_{2}=0.1$, and (**c**) $B_{1}=0.1$ and $B_{2}=1$. The legend for all panels is as shown in panel (**c**).

The dynamical behavior of the next to nearest neighbor (nnn) entanglement $C_{15}$ is depicted in Figure 3. In Figure 3a, $C_{15}$ starts at a zero value which is maintained for a very short period of time that increases as the temperature is raised, then it increases reaching a peak value that decreases with temperature before decaying and maintaining a zero value at all temperatures except zero, where it revives again making a much smaller peak before completely vanishing. The inhomogeneous magnetic field, $B_{2}<B_{1}$, case shows a very similar behavior but with a slightly longer zero period at the beginning and higher peak value, as shown in Figure 3b. Applying an inhomogeneous magnetic field with a higher strength at the central spin leads to a similar behavior as the previous cases but with much lower peak values and much longer zero-entanglement period at the beginning as can be seen in Figure 3c. The entanglement $C_{17}$ was found to maintain a zero value at all times at all magnetic field combinations. The time evolution of the Ising system starting form an initially disentangled, separable, state is presented in Figure 4. The dynamics of $C_{12}$ is depicted in the upper panels of Figure 4, which shows that the entanglement starting at zero value revives monotonically within a finite period of time to reach an asymptotic steady state value in all magnetic field arrangements. The difference between the homogeneous case, in Figure 4a, and the inhomogeneous case, where $B_{1}>B_{2}$, in Figure 4b is quite small, where the steady state values are slightly reduced. However, applying an inhomogeneous field, where $B_{2}>B_{1}$ raises the steady state value significantly and increases robustness against temperature as shown in Figure 4c. Moreover, the zero entanglement period in Figure 4a,b are almost the same but much longer than the one in Figure 4c.

Comparing Figure 2 and Figure 4, one can conclude that the steady state values of $C_{12}$ are exactly the same regardless of the initial state of the system, i.e., the system evolves to the same final state independent of its initial setup, including the partially entangled state, $|\psi_{w}\rangle$, which we have tested as well, but is not presented here. The entanglement $C_{14}$, which is presented in the lower panels of Figure 4, also, revives from zero to a steady state value that depends again on the inhomogeneity of the magnetic field, where as can be seen, the asymptotic value and thermal robustness are much higher in panel (b), where $B_{2}<B_{1}$, compared with panels (a) and (c), where particularly in (c), $C_{14}$ becomes very fragile to the thermal effect. The nnn entanglement $C_{15}$ was found to maintain a zero value at all times at zero and no-zero temperatures, which we do not show here. Again, comparing Figure 2 and Figure 4, shows that the asymptotic value of $C_{14}$ is independent of the initial state of the system.

### 3.2. Partially Anisotropic System (XYZ Model)

**Figure 5:**
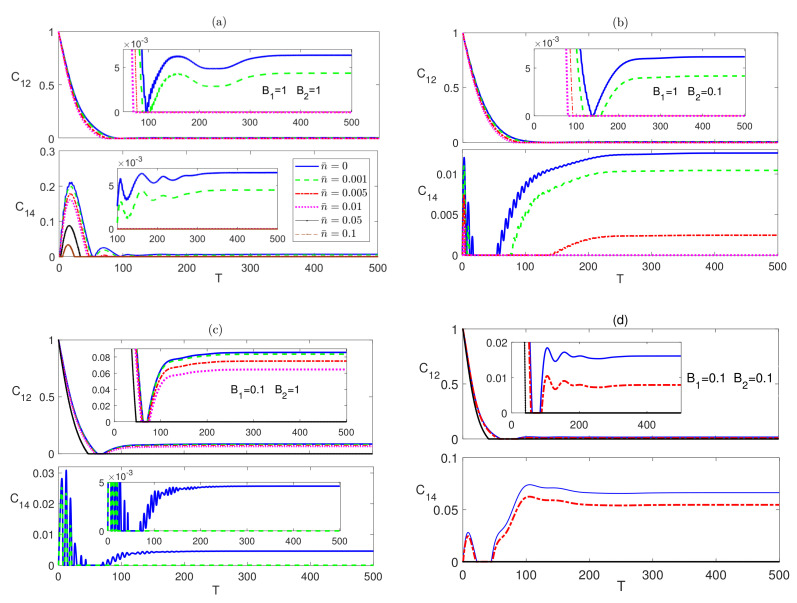
Time evolution of $C_{12}$ and $C_{14}$ in the XYZ system in the presence of the environment (Γ=0.05) starting from an initial maximally entangled state at different temperatures ($0\leq \bar{n}\leq 0.05$) and different magnetic field strengths (**a**) $B_{1}=1$ and $B_{2}=1$, (**b**) $B_{1}=1$ and $B_{2}=0.1$, (**c**) $B_{1}=0.1$ and $B_{2}=1$ and (**d**) $B_{1}=0.1$ and $B_{2}=0.1$. The legend for all panels is as shown in panel (**a**).

**Figure 6:**
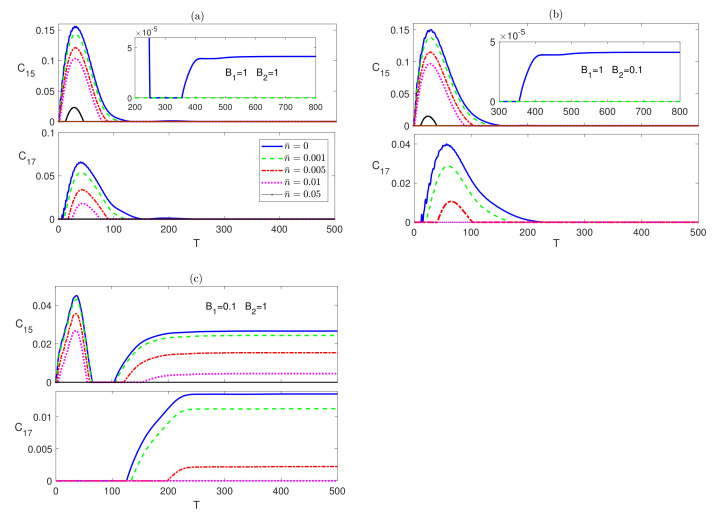
Time evolution of $C_{15}$ and $C_{17}$ in the XYZ system in the presence of the environment (Γ=0.05) starting from an initial maximally entangled state at different temperatures ($0\leq \bar{n}\leq 0.1$) and different magnetic field strengths (**a**) $B_{1}=1$ and $B_{2}=1$, (**b**) $B_{1}=1$ and $B_{2}=0.1$, and (**c**) $B_{1}=0.1$ and $B_{2}=1$. The legend for all panels is as shown in panel (**a**).

Studying the same spin system at a partial degree of anisotropy of the spin-spin interaction shows some similarities to the completely anisotropic (Ising) system but manifests striking differences as well. In this section, we investigate the time evolution of the partially anisotropic (XYZ) Heisenberg system, where γ=1/2 and δ=1. We present the dynamics of the system starting form a maximally entangled state in Figure 5 and Figure 6.

The entanglement $C_{12}$ shows a very similar profile to the corresponding one in Ising case, presented in Figure 2, at all magnetic field setups, as shown in the upper panels of Figure 5. Nevertheless, there is a notable difference in the asymptotic values of $C_{12}$, lowering the anisotropy reduces these values to almost their half magnitude, except in Figure 5c, where there is only a slight decrease in the steady state values. On the other hand, increasing anisotropy has another damaging effect as it reduces the robustness of the system to thermal excitation at all degrees of inhomogeneity of the field as can be concluded from Figure 5a–c compared with Figure 2a–c respectively. The dynamics of $C_{14}$ is plotted in the lower panels of Figure 5, which in a similar fashion to $C_{12}$, shows asymptotic values that are about half the magnitude of the corresponding ones in the Ising system for all cases of magnetic field including the case of $B_{1}<B_{2}$ illustrated in Figure 5c, in contrast to the behavior of $C_{12}$ in that particular case. Applying a homogeneous weak magnetic field to the XYZ system leads to an effect similar to what was observed in the Ising system, as shown in Figure 5d, where the steady state values of both of $C_{12}$ and $C_{14}$ are higher than the corresponding ones in the homogeneous strong magnetic field case but are lower than the corresponding ones in the weak field of the Ising case, shown in Figure 2d. However, the inhomogeneous field effect on the XYZ system as illustrated in Figure 5c, still offers much higher boost to the steady state entanglement of the border sites and higher thermal resistance.

The most distinguished behavior of the XYZ system, compared with the Ising system, manifests itself in the nnn entanglement $C_{15}$ and the nnnn entanglement $C_{17}$, which are depicted in the upper and lower panels of Figure 6 respectively. In contrary to the Ising system case, the nnn entanglement $C_{15}$ does not vanish asymptotically at zero temperature, although it does at non-zero temperatures, where it reaches a very small steady state value in both cases of homogeneous and inhomogeneous magnetic field $\left(B_{1}>B_{2}\right)$, as shown in Figure 6a,b respectively. Interestingly, in the other case of the inhomogeneous magnetic field $\left(B_{1}<B_{2}\right)$, depicted in panel (c), $C_{15}$ shows high robustness against thermal effects and higher asymptotic values that even exceeds that of the nn entanglement $C_{14}$, illustrated in Figure 5c. More interestingly, the nnnn entanglement $C_{17}$, shown in the lower panels of Figure 6c, where $B_{1}<B_{2}$, evolves from zero before reviving and reaching asymptotically a non-zero steady state value at zero and non-zero temperatures, which are also higher than the corresponding $C_{14}$ values illustrated in Figure 5c. The other two cases of magnetic field, shown in Figure 6a,b result in an asymptotically vanishing $C_{17}$. Therefore, applying an inhomogeneous magnetic field to the spin system, where the field gradient is directed inward enhances the entanglement among the border spins, even the nnnn neighbors, and increases its robustness against thermal excitation.

**Figure 7:**
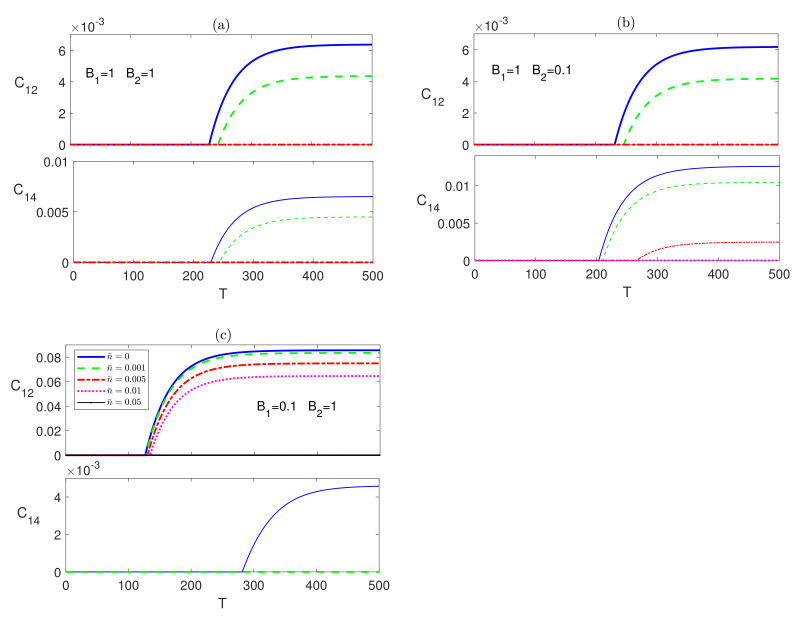
Time evolution of $C_{12}$ and $C_{14}$ in the XYZ system in the presence of the environment (Γ=0.05) starting from an initial disentangled state at different temperatures ($0\leq \bar{n}\leq 0.05$) and different magnetic field strengths (**a**) $B_{1}=1$ and $B_{2}=1$, (**b**) $B_{1}=1$ and $B_{2}=0.1$, and (**c**) $B_{1}=0.1$ and $B_{2}=1$. The legend for all panels is as shown in panel (**c**).

We tested the time evolution of the dissipative XYZ system under the effect of different magnetic field configurations starting from an initial disentangled state. We found that the different entanglements, nn, nnn and nnnn bipartite, start from a zero value before reviving and increasing monotonically to asymptotically reach steady state values that coincide with the corresponding ones in the case of an initial maximally entangled state, as was discussed in Figure 5 and Figure 6, in a very similar fashion to what was observed in the Ising system. In Figure 7, as an illustration, we depict the time evolution of C12 and C14 starting from the disentangled initial state, which shows that $C_{12}$ and $C_{14}$ evolve to reach asymptotically the same steady state values, presented in Figure 5, depending only on the anisotropy of the system, the temperature and the inhomogeneity of the magnetic field. Testing the system dynamics starting from an initial partially entangled state $|\psi_{w}\rangle$, shows that it leads to the same asymptotic state as well. We did not insert the graphs of the comparison of the other cases to save space and avoid redundancy.

### 3.3. Isotropic System (XXX Model)

**Figure 8:**
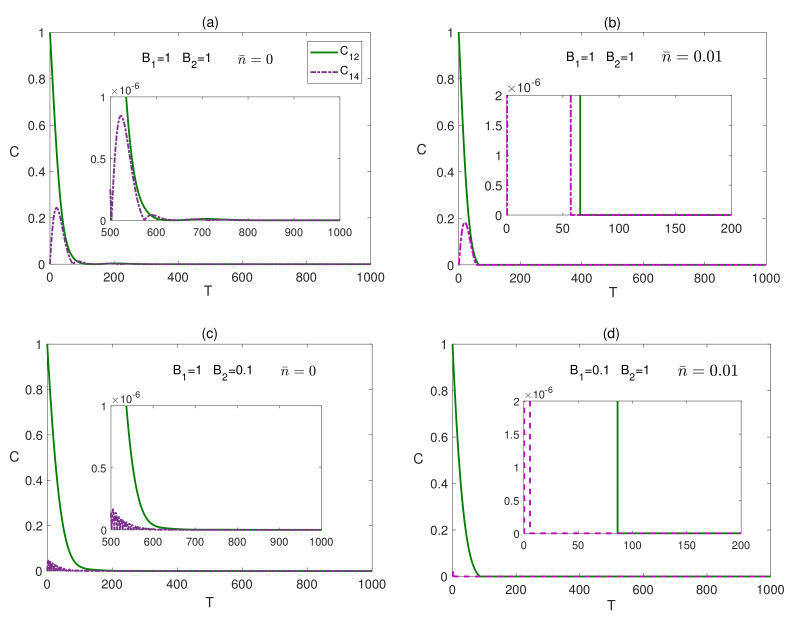
Time evolution of $C_{12}$ and $C_{14}$ in the XXX system in the presence of the environment (Γ=0.05) starting from an initial maximally entangled state, at different temperatures and magnetic fields, where in (**a**) $\bar{n}=0$, $B_{1}=1$ and $B_{2}=1$, (**b**) $\bar{n}=0.01$, $B_{1}=1$ and $B_{2}=1$, (**c**) $\bar{n}=0$, $B_{1}=1$ and $B_{2}=0.1$ and (**d**) $\bar{n}=0.01$, $B_{1}=0.1$ and $B_{2}=1$. The legend for all panels is as shown in panel (**a**).

The time evolution of the bipartite entanglements in a completely isotropic (XXX) system is explored in Figure 8, starting from a maximally entangled state, where the legend in this figure is different from the default one in this paper. In Figure 8a, the dynamics of $C_{12}$ and $C_{14}$ is depicted under the effect of a homogeneous magnetic field at zero temperature. The entanglement $C_{14}$, after displaying an oscillatory behavior vanishes within a finite period of time, while $C_{12}$ decays from a maximum value to zero monotonically within a smaller period of time. Raising the temperature, in the presence of a homogeneous magnetic field, causes a sudden death of entanglement at a much earlier time as illustrated in Figure 8b. The effect of an inhomogeneous magnetic field at different temperatures is shown Figure 8c,d. Applying any inhomogeneous magnetic field, $B_{1}>B_{2}$, at zero temperature, reduces the entanglement oscillation, and the entanglement vanishes within a finite period of time close to that of the homogeneous field case, as illustrated in Figure 8c. Considering the other inhomogeneous field case, at zero temperature, does not show a significant change from what is shown in Figure 8c, but as the temperature is raised, a sudden death behavior is observed where the death times, for $C_{12}$ and $C_{14}$, are quite distinguished from each other, as illustrated in Figure 8d, compared with the homogeneous field case shown in Figure 8b. Testing the XXX system behavior under a weak homogeneous field, $B_{1}=B_{2}=0.1$, shows some changes compared with the case of a strong homogeneous magnetic field, presented in Figure 8a,b. The reviving peaks of the entanglement $C_{12}$ and $C_{14}$ are higher than the corresponding ones in the strong homogeneous magnetic field case with stronger thermal persistence, and the entanglement vanishes eventually after a longer time but shorter than the inhomogeneous magnetic field case particularly for the border sites. However, in all cases, the entanglement in the XXX system vanishes asymptotically regardless of the system setup, where the environment dissipative decay effect dominates over the net magnetic field acting on the spins, aligning all spins down, at zero temperature, or close to down at finite temperature, to a separable steady state, which will be discussed further in the next section. Comparing the behavior of the bipartite entanglement in the triangular lattice, in a homogeneous field, with the one-dimensional system case reported in [37], one can notice a strong similarity in the entanglement dynamics and asymptotic profile, which is not a surprise. In both cases, each spin is driven by the effective mean magnetic field of the other spins while exposed to the dissipative effect of the environment as we explained earlier, where the asymptotic state of the spin and the entanglement steady state are decided by the competitive effects of these two parameters. However, as expected, the different spin configurations lead to different early dynamics and different steady state values.

## 4. Spin Relaxation

### 4.1. Ising System

**Figure 9:**
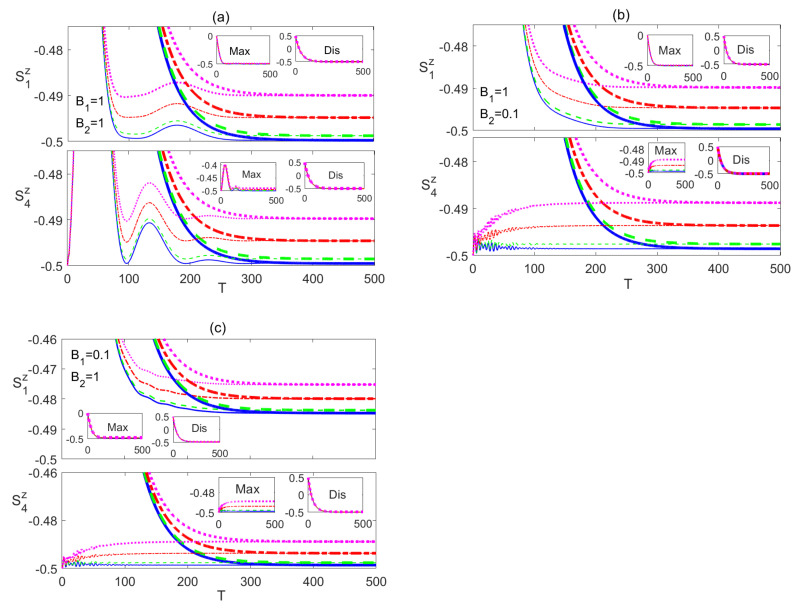
Time evolution of the spin state $\langle S_{1}^{z}\rangle$ and $\langle S_{4}^{z}\rangle$ in the Ising system in the presence of the environment (Γ=0.05) at different temperatures ($0\leq \bar{n}\leq 0.01$), and different magnetic field strengths, where (**a**) $B_{1}=1$ and $B_{2}=1$, (**b**) $B_{1}=1$ and $B_{2}=0.1$, and (**c**) $B_{1}=0.1$ and $B_{2}=1$. The legend for all panels is as shown in Figure 7c. The inner inset plots in the panels in this figure provide an overall look at the spin dynamics presented in the corresponding panels.

It is very important and enlightening to explore how the spin state evolves in time under the different system configurations, compare and correlate it with the time evolution of the corresponding entanglements reported before, particularly their asymptotic behavior. In Figure 9a, we study the time evolution of the spin state at the border site 1 and the central site 4 in the dissipative Ising system in the presence of a homogeneous magnetic field, $B_{1}=B_{2}=1$, at different temperatures starting from different initial states; maximally entangled (thin lines) and disentangled (bold lines).

In the upper panel of Figure 9a, we compare the time evolution of the spin 1 state $\langle S_{1}^{z}\rangle$ starting from two different initial states, maximally entangled and disentangled (separable), represented by $|\psi_{m}\rangle$ and $|\psi_{s}\rangle$ respectively. In the maximally entangled state case, represented by the thin plots, at all temperatures, $\langle S_{1}^{z}\rangle$ starts from zero, decays, reaches a minimum value, then shows a brief oscillation before reaching a steady state value that is very close to −0.5 at zero temperature but increases as the temperature is raised. Obviously, the spin state starts at zero value but asymptotically, under the decay effect of the environment, it is pushed downward; however, due to the impact of the precession motion induced by the net magnetic field, it ends up at a steady state value that is slightly higher than −0.5 at zero temperature. It deviates further up, away from −0.5, as the temperature increases due to the thermal excitation as expected. Starting from a disentangled state value, 0.5, depicted in Figure 9a as bold lines, it decays monotonically reaching a steady state value that coincides with that of the maximally entangled initial state case at all temperatures. The inner inset plots in this figure and all coming figures represent the overall dynamics of the concerned spin state. In fact, comparing the dynamics of the spin state, in the current set up, with the corresponding bipartite entanglements that we reported in Figure 2, Figure 3 and Figure 4, one can notice a strong resemblance. The entanglements corresponding to the maximally entangled state show oscillatory behavior before reaching a steady state value, while that starting from a disentangled state increases from zero monotonically before reaching the same steady state asymptotically. In addition, the time rate to reach the steady state is very much the same for the spin state and the entanglements.

In the lower panel of Figure 9a, we explore the time evolution of the state of the central spin, 4, in the Ising system starting from the maximally entangled state (thin lines) and the disentangled one (bold lines). As can be noticed, the dynamics of $\langle S_{4}^{z}\rangle$ shows a very similar behavior to that of $\langle S_{1}^{z}\rangle$, which depends on the initial state but it reaches asymptotically a common steady state value regardless of the initial state at all temperatures. Comparing the behavior of $\langle S_{1}^{z}\rangle$ and $\langle S_{4}^{z}\rangle$, starting from the initial maximally entangled state, shows that they reach their steady state values at around the same time but these values are not coinciding as can be seen, the value of $\langle S_{4}^{z}\rangle$ is slightly higher than that of $\langle S_{1}^{z}\rangle$. This behavior should be expected as the central spin is interacting with more nearest-neighbor spins compared with the border spin, which provides a stronger precession and consequently a stronger resistance to the environment decay effect. The effect of an inhomogeneous magnetic field on the time evolution of the spin states of the Ising system is explored in Figure 9b, where $B_{1}>B_{2}$. The dynamics of $\langle S_{1}^{z}\rangle$ is depicted in the upper panel of Figure 9b starting from a maximally entanglement state (thin lines) and a disentangled state (bold lines), which shows a very similar behavior to what was observed in the homogeneous case except that for both of the initial states, the spin state decays monotonically without any oscillation, reaching a common steady state value at each temperature, which is very slightly higher than that of the homogeneous field case. Therefore, although the magnetic field at the boarder sites is still of the same strength, applying a weaker field at the center deviates the asymptotic value further away from −0.5. The dynamics of $\langle S_{4}^{z}\rangle$ is plotted in the lower panel of Figure 9b, where it starts at −0.5 for the maximally entangled state (thin lines) and 0.5 for the disentangled state (bold lines) but in both cases, it evolves to a common steady state value. Comparing the lower panel of Figure 9b with the upper panel, one can notice an increase in the steady state values of $\langle S_{4}^{z}\rangle$ at all temperatures as a result of applying a weaker magnetic field at the central spin. Comparing the dynamics of $\langle S_{1}^{z}\rangle$ and $\langle S_{4}^{z}\rangle$ and their asymptotic steady state values starting form a disentangled state, where both spins are initially pointing upward with the same value 0.5. The steady state value of $\langle S_{4}^{z}\rangle$ is clearly higher than that of $\langle S_{1}^{z}\rangle$, at all temperatures, as a result of the applied inhomogeneous magnetic field. The difference in the steady state values is larger than what was observed in the homogeneous magnetic field case discussed in Figure 9a.

The other inhomogeneous magnetic field case, where $B_{1}=0.1$ and $B_{2}=1$, is presented in Figure 9c. The common steady state values of $\langle S_{1}^{z}\rangle$, as illustrated in the upper panel of Figure 9c, is much higher than that was reported in the previous cases in Figure 9a,b, as a result of the weak magnetic field strength applied at the border sites in the current case. On the other hand, the common steady state values of $\langle S_{4}^{z}\rangle$, shown in the lower panel of Figure 9c, is slightly lower than what was observed in the lower panels of Figure 9a,b, which emphasizes the very small impact of varying the magnetic field strength on the central spin. Comparing the upper and the lower panels of Figure 9c shows a huge difference between the corresponding steady state values of $\langle S_{1}^{z}\rangle$ and $\langle S_{4}^{z}\rangle$, compared with what was observed in the two previous magnetic fields configurations reported in Figure 9a,b. This shows that in this particular configuration, $\langle S_{1}^{z}\rangle$ and $\langle S_{4}^{z}\rangle$ end up in two completely different states and the steady state of $\langle S_{1}^{z}\rangle$ deviates the most away from the downward state, in contrrast to $\langle S_{4}^{z}\rangle$, which an indication that the net magnetic field in this case is significantly enhancing the spin precession motion against the dissipative decay effect, causing the bipartite entanglement, as well as the robustness against thermal effects, to be considerably boosted, as was illustrated Figure 2c and Figure 4c.

### 4.2. XYZ System

**Figure 10:**
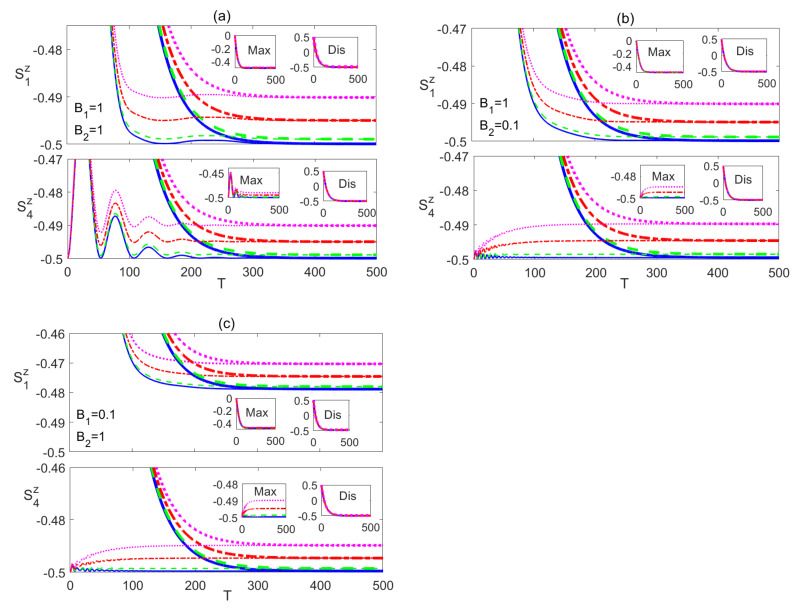
Time evolution of the spin state $\langle S_{1}^{z}\rangle$ and $\langle S_{4}^{z}\rangle$ in the XYZ system in the presence of the environment (Γ=0.05) at different temperatures ($0\leq \bar{n}\leq 0.01$), and different magnetic field strengths, where (**a**) $B_{1}=1$ and $B_{2}=1$, (**b**) $B_{1}=1$ and $B_{2}=0.1$, and (**c**) $B_{1}=0.1$ and $B_{2}=1$. The legend for all panels is as shown in Figure 7c. The inner inset plots in the panels in this figure provide an overall look at the spin dynamics presented in the corresponding panels.

The spin state dynamics of the dissipative partially anisotropic (XYZ) spin system under different magnetic field configurations is explored in Figure 10. Comparing Figure 10a with Figure 9a, where the magnetic field is homogeneous, one can see that $\langle S_{1}^{z}\rangle$ and $\langle S_{4}^{z}\rangle$ in the partially anisotropic system behave in a very similar way to the Ising system case except that the oscillatory behavior is reduced in the XYZ system and the asymptotic values, at all temperatures, are slightly lower than that of the Ising case, which is expected as the effect of the spin-spin interaction in the current case is lower than the anisotropic case. Turning to the inhomogeneous magnetic field case, where $B_{1}>B_{2}$, comparing Figure 10b and Figure 9b, it shows that, again, the dynamic behavior of $\langle S_{1}^{z}\rangle$ and $\langle S_{4}^{z}\rangle$ is very similar to the Ising case, but the asymptotic equilibrium values are lower even compared with the homogeneous field case.

The interesting change takes place in the other inhomogeneous field case, $B_{1}<B_{2}$, presented in Figure 10c, where the magnetic field strength at the border sites is much smaller than the one at the central site. While there is no notable change in the $\langle S_{4}^{z}\rangle$ behavior compared with that depicted in Figure 10b, the steady state values of $\langle S_{1}^{z}\rangle$ are much higher than the corresponding ones in the Ising case, illustrated in Figure 9b. One may have expected a different behavior for $\langle S_{1}^{z}\rangle$, and all the border spins, where they should have relaxed asymptotically to a steady state value that is lower, closer to the downward state, than the corresponding one of the Ising system, similar to the two previous magnetic field configurations, illustrated in Figure 10a,b. However, the observed behavior means that the precession motion of the border spins is enhanced and persistent against the dissipative decay effect and thermal excitation, which may explain the strong long range, beyond nearest neighbors, entanglement observed in this system configuration, as illustrated in Figure 6c. This also might be a sign of a critical behavior of the system at this particular combination of the system parameters that needs further investigation.

### 4.3. XXX System

**Figure 11:**
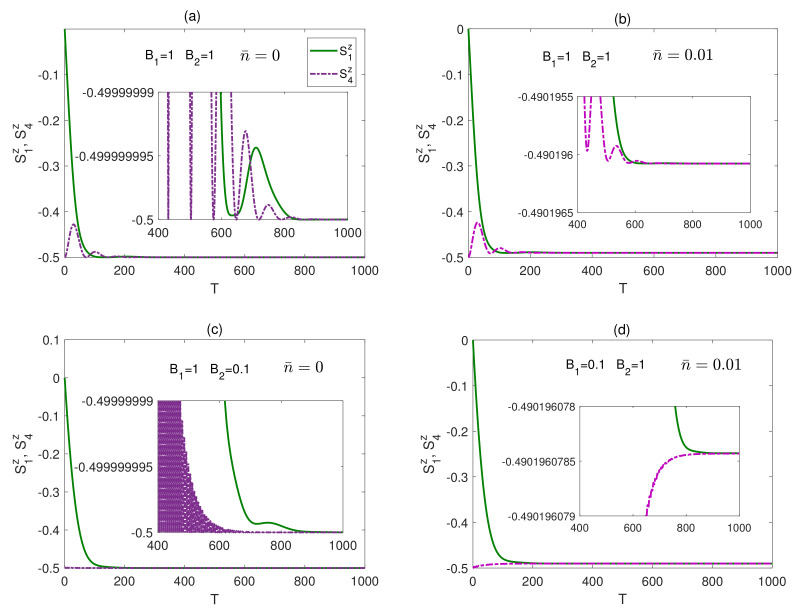
Time evolution of $\langle S_{1}^{z}\rangle$ and $\langle S_{4}^{z}\rangle$ in the XXX system in the presence of the environment (Γ=0.05) starting from an initial maximally entangled state, at different temperatures and magnetic fields, where in (**a**) $\bar{n}=0$, $B_{1}=1$ and $B_{2}=1$, (**b**) $\bar{n}=0.01$, $B_{1}=1$ and $B_{2}=1$, (**c**) $\bar{n}=0$, $B_{1}=1$ and $B_{2}=0.1$ and (**d**) $\bar{n}=0.01$, $B_{1}=0.1$ and $B_{2}=1$. The legend for all panels is as shown in panel (**a**). The inner inset plots in the panels in this figure provide a magnifying look at the asymptotic behavior of the spin dynamics presented in the corresponding panels.

We study here the time evolution of the spin state in the completely isotropic dissipative (XXX) system, where a representative sample of the results are depicted in Figure 11. We found that, at zero temperature regardless of the magnetic field configuration or the initial state, the steady state of both of $\langle S_{1}^{z}\rangle$ and $\langle S_{4}^{z}\rangle$ takes exactly the value −0.5, which as we discussed before is due to the fact that the spins are pointing along the net magnetic field direction with no precession motion at all and as a result the dissipative decay effect dominates and force all the spins to, eventually, align downward, parallel to each other reaching a final separable (disentangled) state, as shown in Figure 11a,c. At a non-zero temperature, the spin states $\langle S_{1}^{z}\rangle$ and $\langle S_{4}^{z}\rangle$, due to thermal excitation, relax to a common final steady state value that is higher than −0.5, where the higher the temperature is, the larger is the deviation, as shown in Figure 11b. Applying an inhomogeneous magnetic field does not split the common steady state value of $\langle S_{1}^{z}\rangle$ and $\langle S_{4}^{z}\rangle$, even at non-zero temperature as can be noticed in Figure 11d. This means, in the XXX system case, all the spins end up pointing in the same direction reaching a separable disentangled steady state, as was pointed out in Figure 8, regardless of the initial state or the system parameters.

## 5. Conclusions

We studied a finite two-dimensional Heisenberg spin lattice with nearest-neighbor spin interaction coupled to a dissipative Lindblad environment in the presence of an external inhomogeneous magnetic field at finite temperature. The spin lattice consists of a central spin surrounded by 6 border spins equally distant form it in a triangular symmetric structure. We developed an exact numerical solution for the Lindblad master equation of the system, under the Born-Markovian constrain, in Liouville space. We have shown that applying an inhomogeneous magnetic field, compared with the homogeneous one, has a great impact on the entanglement distribution among the spins in the lattice and can be used to significantly enhance the bipartite entanglement among the spins in the system, even beyond nearest neighbors, and boost their thermal robustness at different degrees of anisotropy. In particular, applying an inhomogeneous magnetic field with a gradient directed inward, where the central spin is exposed to higher magnetic field strength compared with the border spins, has the most significant impact on the entanglement enhancement and robustness against the thermal dissipative environment at all degrees of anisotropy, compared with the other magnetic field configurations. Applying such a field to a completely anisotropic (Ising) system, enhanced the nearest neighbor entanglement among the border spins in the steady state considerably, though the bipartite entanglements involving the central spin were slightly reduced. All the beyond nearest neighbor bipartite entanglements of all spins vanish asymptotically in this setup. However, when the same inhomogeneous field was applied to a partially anisotropic (XYZ) system, not only it has significantly enhanced the steady state entanglement among the nearest neighbor border spins, and its thermal robustness, but also among all the beyond nearest neighbor spins in a remarkable way, which indicates that this combination of inhomogeneous external magnetic field and anisotropic spin-spin interaction creates a long range quantum correlation across the lattice. The entanglement in the isotropic (XXX) system was found to asymptotically vanish regardless of the initial state of the system, the temperature or the degree of inhomogeneity of the magnetic field, where the last affects only the length of the time period the system spends before completely losing its entanglement as well as the loss rate. Testing the effect of a weak homogeneous magnetic field on the system at different degrees of anisotropy showed that the entanglement among the different spins is enhanced compared with the strong homogeneous magnetic field case with higher thermal persistence. However, the effect of the inward inhomogeneous magnetic field was still much higher on the entanglement steady state value and thermal robustness of the system at all degrees of anisotropy.

Furthermore, we investigated the spin state dynamics in the system and its correlation to the entanglement behavior under the different system configurations. We have demonstrated that in the isotropic (XXX) system, the dissipative decay effect dominates entirely, over the other system parameters influences, forcing all the spins to align parallel to each other, downward at zero temperature or very slightly away from the downward state at finite temperature, into a separable (disentangled) steady state, regardless of the system initial state or the inhomogeneity of the magnetic field. On the other hand, for anisotropic system, the inhomogeneous magnetic field, with again an inward gradient, was found to have the greatest impact on the spins dynamics and steady state. The complete anisotropy, in the Ising system, enhanced the system robustness against the environment thermal dissipative decay effect, protecting the mutual entanglement among the spins and causing them to asymptotically relax to different steady states that depend on their locations in the lattice, deviating from the downward state induced by the decay. Interestingly, the XYZ system, where the degree of anisotropy is lower than that of the Ising system, exhibited a stronger robustness to the environment decay effect, which was reflected in more distinguished steady states of the spins, from each other, and further away from the downward state. These states, as we have already pointed out, were accompanied by a long range quantum entanglement among the spins across the lattice, which is a sign of a critical behavior of the system taking place at this configuration.

## Data Availability

The data presented in this study are available on request from the corresponding author.
